# Transoral traumatic perforation of the pyriform sinus by a marker pen: report of an infant case

**DOI:** 10.1186/s40792-023-01781-x

**Published:** 2023-11-16

**Authors:** Noboru Oyachi, Fuminori Numano, Tomohiro Saito, Minako Hoshiai, Keiichi Koizumi

**Affiliations:** 1 Department of Pediatric Surgery, Yamanashi Prefectural Central Hospital, 1-1-1 Kofu, Yamanashi, 409-8506 Japan; 2 Department of Pediatrics, Yamanashi Prefectural Central Hospital, 1-1-1 Kofu, Yamanashi, 409-8506 Japan

**Keywords:** Pyriform sinus, Perforation, Hypopharyngeal trauma, Pharyngoesophagography, Antibiotic therapy, Drainage, Infant

## Abstract

**Background:**

Perforation of the pyriform sinus, included in hypopharyngeal injury, is a rare condition typically caused by iatrogenic factors. We present a case of an infant who developed deep cervical and mediastinal abscesses due to a traumatic pyriform sinus perforation caused by accidentally falling with a marker pen in the mouth.

**Case presentation:**

An 11-month-old healthy male infant fell on a trampoline with a marker pen in his mouth. The patient developed swelling in the neck 3 h after the incident and was taken to a regional general hospital. Although a laryngoscopy showed no perforation in the oral cavity or posterior pharynx, a computed tomography (CT) scan revealed significant emphysema extending from the cervix to the mediastinum. The patient was transferred to our tertiary hospital and admitted to the intensive care unit, where he was mechanically ventilated, and antibiotic therapy was initiated. On day 3 of admission, a CT scan revealed deep abscesses in the cervical and upper posterior mediastinum with pneumomediastinum. Although his respiratory status stabilized and he was temporarily weaned, the fever recurred. Pharyngoesopagography revealed significant leakage of contrast from the left pyriform sinus to the mediastinum. Consequently, surgical drainage of the abscess was performed on day 10. Two low-pressure continuous suction drains were placed, one in the posterior mediastinum and the other close to the pyriform sinus. Pharyngoesophagography on postoperative day (POD) 7 demonstrated decreased contrast leakage into the posterior mediastinum. The patient was initiated on enteral nutrition through a nasogastric tube. The patient was discharged on POD 31 after the suction drains were replaced with open Penrose drains, and enteral nutrition via nasogastric tube was continued at home. The Penrose drains were removed on POD 54, and salivary leakage ceased on POD 111.

**Conclusions:**

Although injuries to the oral cavity and posterior pharynx are more easily recognized, the existence of injury in the pyriform sinus can be challenging to evaluate. However, prompt and appropriate management, including intubation, antibiotic therapy, surgical drainage, and nutritional support, is critical in preventing life-threatening complications.

## Introduction

Perforation of the pyriform sinus, part of hypopharyngeal trauma, is a rare condition typically caused by iatrogenic factors, including endotracheal intubation, esophagoscopy, and nasogastric tube placement in all age groups [1]. Injuries to the oral cavity and posterior pharyngeal wall are common in children who fall with stick-shaped foreign objects, such as toothbrushes, spoons, pencils, or chopsticks in their mouths. However, specific injuries to the pyriform sinus are infrequent [2, 3].

We present a case of an infant who developed deep cervical and mediastinal abscesses due to a traumatic pyriform sinus perforation caused by accidentally falling with a marker pen in the mouth.

## Case presentation

**Fig. 1:**
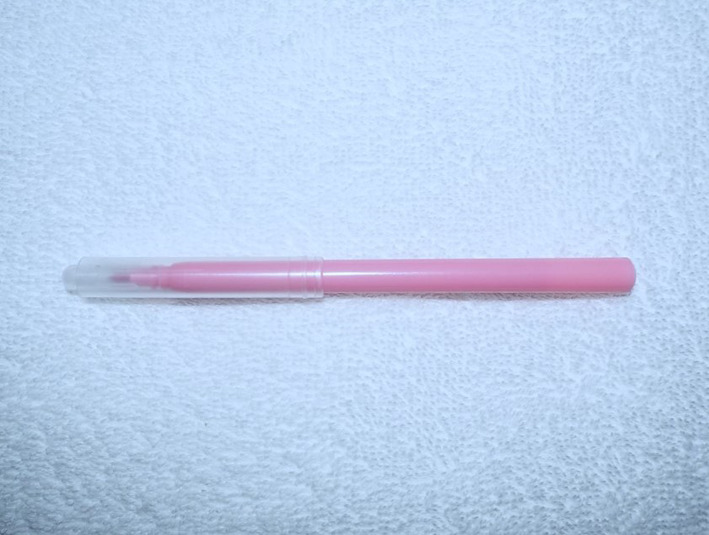
Marker pen that was in the patient’s mouth when he fell on a trampoline

We describe the case of an 11-month-old healthy male who was found crying by his parents after falling on a trampoline with a marker pen in his mouth (Fig. 1). A small amount of bleeding was observed in the mouth, and there was no evidence of impaired consciousness or respiratory distress. A local dentist and an otolaryngologist initially examined the patient, and he was sent home as no specific findings were noted. Although the patient had not ingest anything orally, he developed swelling in the neck 3 h after the incident, and was taken to a regional general hospital.

**Fig. 2:**
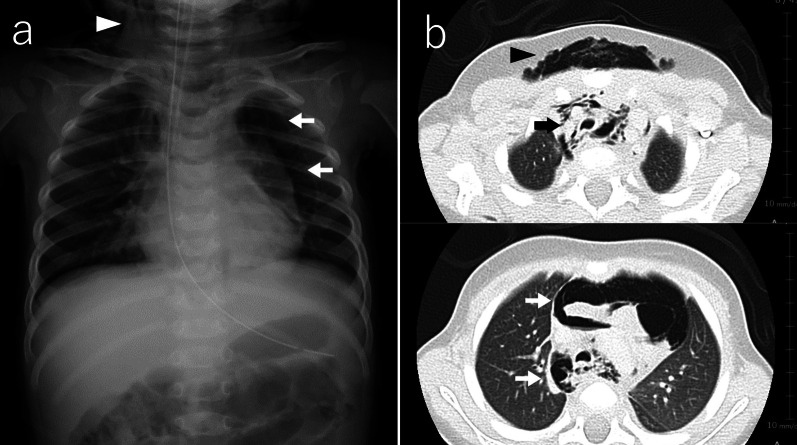
Chest X-ray (**a**) and a CT scan (**b**) on admission. A chest X-ray and a CT scan revealed extensive subcutaneous emphysema extending in the neck (arrow) and the pneumomediastinum (arrowhead)

Laryngoscopy showed no evidence of perforation in the oral cavity or posterior pharynx. However, a computed tomography (CT) scan showed significant emphysema extending from the cervix to the mediastinum, suggesting an injured airway (Fig. 2), and the patient was immediately transferred to our tertiary hospital.

On admission, vital signs revealed a temperature of 38.8 ℃, blood pressure of 85/60 mm Hg, and a heart rate of 180 bpm. Hematology revealed a white blood cell count of 6800/mm^3^ and a C-reactive protein level of 4.7 mg/dL.

**Fig. 3:**
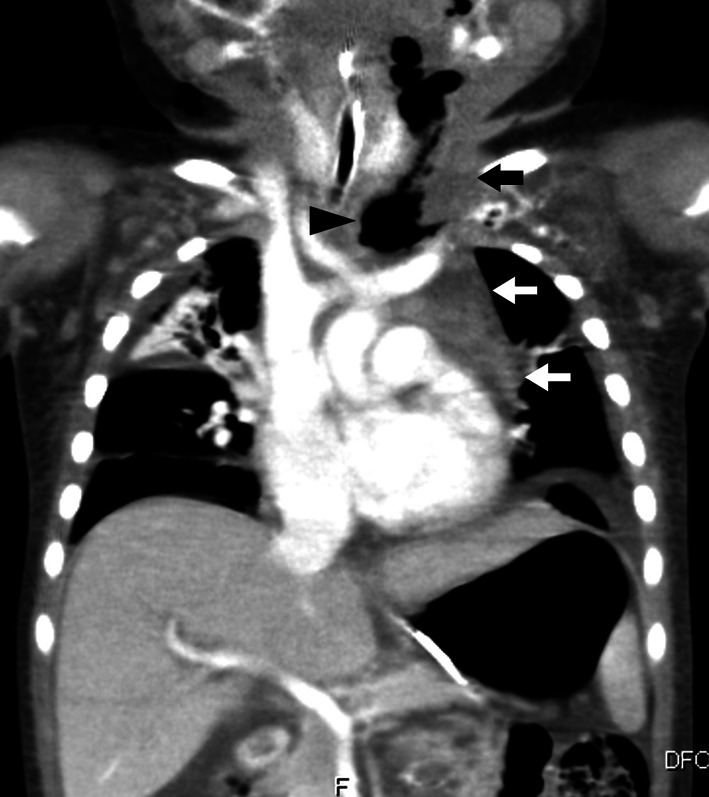
Chest CT scan on day 3 of admission. A CT scan confirmed the presence of deep abscesses (arrow) in the cervical and upper posterior mediastinum with pneumomediastinum (arrowhead)

**Fig. 4:**
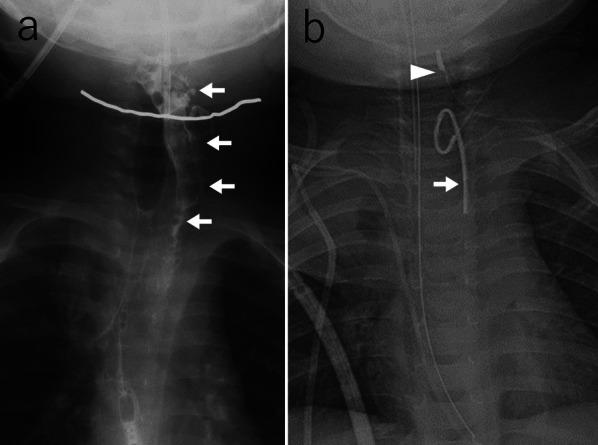
Pharyngoesophagography (**a**) and surgical drainage of the abscess (**b**) performed on day 10. **a** Pharyngoesophagography revealed contrast leakage from the left pyriform sinus into the mediastinum (arrow). **b** Two low-pressure continuous 15 Fr suction drains were placed, one in the posterior mediastinum (arrow) and the other near the pyriform sinus (arrowhead)

The patient was admitted to the intensive care unit, where he was mechanically ventilated, and antibiotic therapy with ampicillin/sulbactam and clindamycin was initiated. On day 3 of admission, a chest X-ray showed an enlarged mediastinal shadow, and a CT scan revealed deep abscesses in the cervical and upper posterior mediastinum with pneumomediastinum (Fig. 3). His respiratory status was stabilized and he was weaned from the ventilator and transferred to a general ward on day 8, but the fever recurred on the same day. A CT scan showed no improvement in the accumulating abscess, and pharyngoesophagography performed on day 10 revealed contrast leakage from the left pyriform sinus into the mediastinum (Fig. 4a). Then, the antibiotic therapy was changed to meropenem and vancomycin regimens.

Surgical drainage of the abscesses was performed on the same day. A 3 cm incision was made on the anterior neck's left side, and the superficial cervical muscles were dissected along the midline due to significant adhesions in the surrounding tissue. The dissection was performed on the left side at the deep level of the sternohyoid muscle and advanced in a cephalad direction along the posterior side of the internal carotid artery and the jugular vein until the abscess cavity was reached. The cavity was found to be continuous from the pharynx to the mediastinum with discharge of pus and saliva. Two low-pressure continuous 15 Fr suction drains were placed: one in the posterior mediastinum and the other close to the pyriform sinus (Fig. 4b).

The postoperative antibiotic therapy was deescalated to ampicillin–sulbactam again on the postoperative day (POD) 3 and continued until 2 weeks postoperatively. Although pharyngoesophagography showed significant contrast leakage from the perforation site, the amount of contrast descending into the posterior mediastinum gradually decreased by POD 7 owing to continuous low-pressure suctioning. Based on these findings, the patient was initiated on enteral nutrition through a nasogastric tube with great caution, albeit relatively late. A CT scan on POD 13 showed a reduced abscess cavity, and pharyngoesophagography performed on POD 20 revealed the formation of a fistula from the abscess cavity to the skin. Consequently, the suction drains were replaced with open Penrose drains. With minimal leakage, the patient was discharged on POD 31, and management with the Penrose drains and enteral nutrition through a nasogastric tube continued at home.

**Fig. 5:**
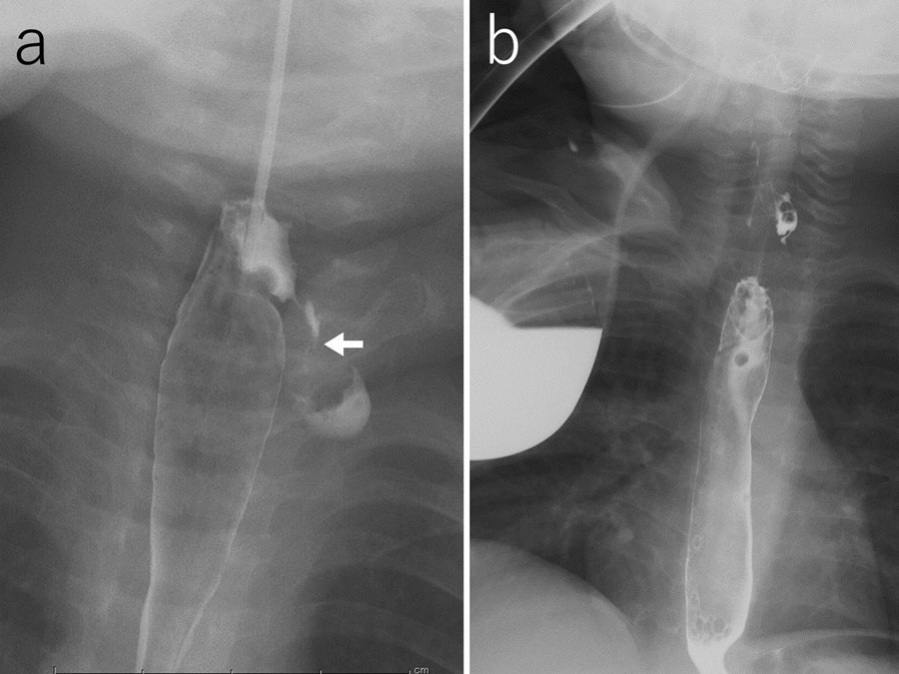
Pharyngoesophagography on POD 54 (**a**) and 117 (**b**). **a** Although a small amount of contract flowed from the fistula (arrow), there was no need for reinsertion of the drains. **b** Contrast leakage ceased from the pyriform sinus

The Penrose drains were removed on POD 54 (Fig. 5a), and salivary leakage completely ceased on POD 111. Tube feeding was discontinued, and the patient began oral feeding on POD 117 (Fig. 5b). His oral intake status was unproblematic, and his development was satisfactory.

## Discussion

Pharyngeal injuries can cause deep cervical abscesses in the retropharyngeal space and severe life-threatening complications, such as acute mediastinitis via the prevertebral space [4, 5]. Other life-threatening complications may occur depending on the location and depth of the injury. The introduction of a foreign body posteriorly into the pharynx risks intracranial injury and caution is also required in diagnosing internal carotid or jugular vein injury if the object is introduced laterally [6].

The unique feature of this case is that the patient fell while playing with a marker pen in the mouth, which perforated the pyriform sinus in the hypopharynx, communicating with the posterior pharyngeal space. The perforation site was not clearly identified on initial examination but was eventually identified by pharyngoesophagography. Moreover, the lesion extended into the mediastinum, requiring temporary mechanical ventilation, and was eventually managed by cervical drainage and antibiotic therapy.

The morphological characteristics of the oropharyngeal region in infants and children indicate that the mid-pharyngeal region between the oral cavity and the esophagus is significantly shorter than in adults, which increases the risk of foreign bodies entering the pharynx from the oral cavity. In addition, the pharynx in children is straighter than that in adults. When evaluating oral trauma, it is critical to consider these morphologic features to recognize the increased likelihood of rod-shaped foreign bodies passing through the oropharynx and reaching the pyriform sinus, potentially causing injury [7, 8]. The retropharyngeal space extends from the 6th cervical vertebra to the 3rd thoracic vertebra, and the posterior space continues to the diaphragm. Therefore, perforation of the pharynx can lead to contamination of the mediastinum and eventually reach the upper surface of the diaphragm, causing acute mediastinitis and mediastinal abscess.

In the case presented, bleeding had ceased at the presentation time, and the laryngoscopy revealed no apparent signs of perforation. However, a CT scan revealed extensive pneumomediastinum from the cervical region. These findings led to early intensive care for the patient. Although the oral cavity and posterior pharyngeal wall can often be visualized, identifying deep injuries in the pyriform sinus can be challenging with laryngoscopy. When deep and severe mediastinitis develops, nonspecific symptoms often manifest within hours, including high fever, cough, chest pain, and dysphagia, followed by progressive dyspnea. In this case, the mucosa of the wound acted as a check valve, and the pneumomediastinum gradually widened due to crying. In cases of discrepancies between the laryngoscopy findings and the patient's symptoms, prompt pharyngoesophagography is necessary [9].

The following management strategy was considered in this case. The patient was intubated and placed under deep sedation for ventilatory management. Although mechanical ventilation is not always necessary in a pneumomediastinum due to pharyngeal trauma, it was deemed necessary in this case, because the lesion was significantly enlarged and at high risk of further expansion.

Antibiotic therapy with or without surgical drainage is the primary treatment [2, 3]. Acute suppurative mediastinitis resulting from pharyngeal injury often involves mixed aerobic and anaerobic bacterial infections, requiring broad-spectrum antibiotic selection. As abscess formation progresses, effective drainage and antibiotic administration become critical. In the present case, the abscesses were localized in the upper posterior mediastinum and deep cervical space and could be drained from the cervical area. In addition, reports explicitly recommend continuous suction with the drain [10], and we also applied continuous suction during the acute phase. However, when antibiotic therapy and surgical drainage fail to improve the condition, using fascial flaps to cover the perforated area can be considered a life-saving intervention. There are several options for fascial flap use [11], and the most appropriate method is chosen on a case-by-case basis. Despite the anticipated large injury site, outpatient enteral nutrition management via a nasogastric tube was deemed feasible. The patient's overall condition was stable, and since reliable drainage management and enteral nutrition could be achieved in the outpatient setting, further invasive surgical intervention was deemed unnecessary.

Raising parental awareness of the risk of complications associated with pharyngeal injuries is critical, and medical professionals should be aware that early recognition and prompt intervention can improve patient outcomes [2, 5, 6]. These incidents are notably reported in children aged 1–2 years, especially as they begin to walk independently. Infants are naturally curious and often explore their environment by putting objects in their mouths. Although products for younger children display warnings about throat-stabbing dangers, there is still insufficient awareness of the potentially fatal consequences of foreign body ingestion.

Pharyngeal trauma should be considered a possibility of abuse if it occurs at an unnatural age or if other physical abnormalities are present. In cases of abuse, there may be a delay in seeking medical attention [12]. It is crucial to consider the possibility of abuse if there is a significant delay between the injury and seeking medical attention.

## Conclusion

We encountered a case of an 11-month-old infant who developed deep cervical and mediastinal abscesses due to a traumatic pyriform sinus perforation caused by an accidental fall with a marker pen in the mouth. Although injuries to the oral cavity and posterior pharynx are easily recognized, the existence of injury in the pyriform sinus can be challenging to evaluate. However, prompt and appropriate management, including intubation, antibiotic therapy, surgical drainage, and nutritional support, is critical in preventing life-threatening complications.

## Data Availability

The data set supporting the conclusion of this article is included within the article.

## Abbreviations

CT: Computed tomography
POD: Postoperative day
